# Sodium-based paracetamol: impact on blood pressure, cardiovascular events, and all-cause mortality

**DOI:** 10.1093/eurheartj/ehad535

**Published:** 2023-08-23

**Authors:** Shishir Rao, Milad Nazarzadeh, Dexter Canoy, Yikuan Li, Jing Huang, Mohammad Mamouei, Gholamreza Salimi-Khorshidi, Aletta E Schutte, Bruce Neal, George Davey Smith, Kazem Rahimi

**Affiliations:** Deep Medicine, Oxford Martin School, University of Oxford, 34 Broad St, Oxford, OX1 3BD Oxfordshire, UK; Nuffield Department of Women's & Reproductive Health, University of Oxford, Women's Centre (Level 3), John Radcliffe Hospital, Oxford, OX3 9DU Oxfordshire, UK; Deep Medicine, Oxford Martin School, University of Oxford, 34 Broad St, Oxford, OX1 3BD Oxfordshire, UK; Nuffield Department of Women's & Reproductive Health, University of Oxford, Women's Centre (Level 3), John Radcliffe Hospital, Oxford, OX3 9DU Oxfordshire, UK; Population Health Sciences Institute, Newcastle University, Newcastle, UK; Deep Medicine, Oxford Martin School, University of Oxford, 34 Broad St, Oxford, OX1 3BD Oxfordshire, UK; Nuffield Department of Women's & Reproductive Health, University of Oxford, Women's Centre (Level 3), John Radcliffe Hospital, Oxford, OX3 9DU Oxfordshire, UK; Deep Medicine, Oxford Martin School, University of Oxford, 34 Broad St, Oxford, OX1 3BD Oxfordshire, UK; Department of Occupational and Environmental Health Sciences, School of Public Health, Peking University, Beijing, China; Deep Medicine, Oxford Martin School, University of Oxford, 34 Broad St, Oxford, OX1 3BD Oxfordshire, UK; Nuffield Department of Women's & Reproductive Health, University of Oxford, Women's Centre (Level 3), John Radcliffe Hospital, Oxford, OX3 9DU Oxfordshire, UK; Deep Medicine, Oxford Martin School, University of Oxford, 34 Broad St, Oxford, OX1 3BD Oxfordshire, UK; Nuffield Department of Women's & Reproductive Health, University of Oxford, Women's Centre (Level 3), John Radcliffe Hospital, Oxford, OX3 9DU Oxfordshire, UK; The George Institute for Global Health, University of New South Wales, Sydney, New South Wales, Australia; The George Institute for Global Health, University of New South Wales, Sydney, New South Wales, Australia; Medical Research Council Integrative Epidemiology Unit (IEU), Bristol Medical School, University of Bristol, Bristol, UK; National Institute for Health Research Bristol Biomedical Research Centre, University Hospitals Bristol NHS Foundation Trust and University of Bristol, University of Bristol, Bristol, UK; Deep Medicine, Oxford Martin School, University of Oxford, 34 Broad St, Oxford, OX1 3BD Oxfordshire, UK; Nuffield Department of Women's & Reproductive Health, University of Oxford, Women's Centre (Level 3), John Radcliffe Hospital, Oxford, OX3 9DU Oxfordshire, UK; Population Health Sciences Institute, Newcastle University, Newcastle, UK

**Keywords:** Paracetamol, Deep learning, Cardiovascular diseases, Hypertension, Blood pressure

## Abstract

**Background and Aims:**

Effervescent formulations of paracetamol containing sodium bicarbonate have been reported to associate with increased blood pressure and a higher risk of cardiovascular diseases and all-cause mortality. Given the major implications of these findings, the reported associations were re-examined.

**Methods:**

Using linked electronic health records data, a cohort of 475 442 UK individuals with at least one prescription of paracetamol, aged between 60 and 90 years, was identified. Outcomes in patients taking sodium-based paracetamol were compared with those taking non–sodium-based formulations of the same. Using a deep learning approach, associations with systolic blood pressure (SBP), major cardiovascular events (myocardial infarction, heart failure, and stroke), and all-cause mortality within 1 year after baseline were investigated.

**Results:**

A total of 460 980 and 14 462 patients were identified for the non–sodium-based and sodium-based paracetamol exposure groups, respectively (mean age: 74 years; 64% women). Analysis revealed no difference in SBP [mean difference −0.04 mmHg (95% confidence interval −0.51, 0.43)] and no association with major cardiovascular events [relative risk (RR) 1.03 (0.91, 1.16)]. Sodium-based paracetamol showed a positive association with all-cause mortality [RR 1.46 (1.40, 1.52)]. However, after further accounting of other sources of residual confounding, the observed association attenuated towards the null [RR 1.08 (1.01, 1.16)]. Exploratory analyses revealed dysphagia and related conditions as major sources of uncontrolled confounding by indication for this association.

**Conclusions:**

This study does not support previous suggestions of increased SBP and an elevated risk of cardiovascular events from short-term use of sodium bicarbonate paracetamol in routine clinical practice.

## Structured Graphical Abstract

**Structured Graphical Abstract:**
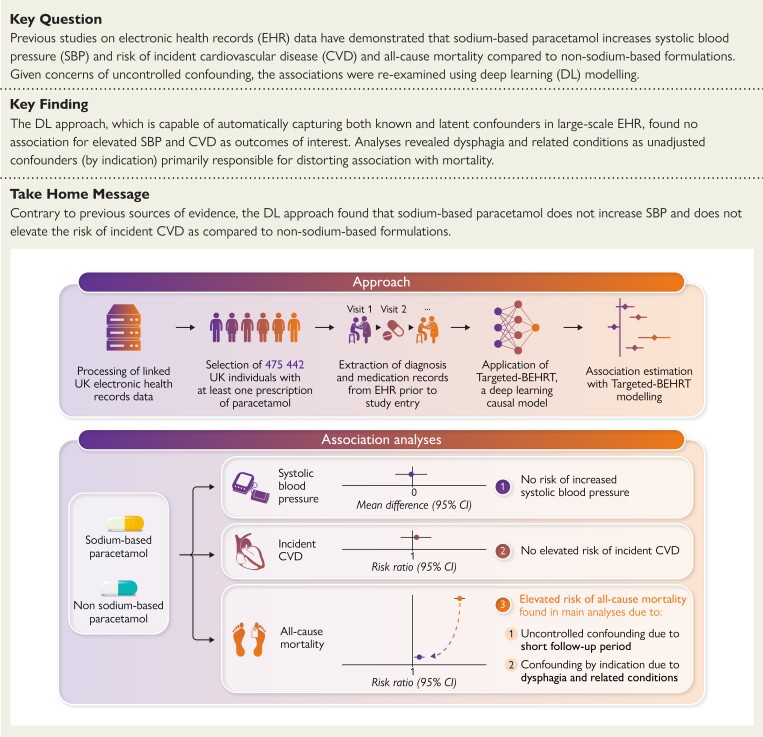
The ‘Approach’ panel presents the cohort selection process and the modelling approach utilized in this study. The ‘Association analyses’ panel presents the findings of the observational analyses. For each outcome, the representational forest plot and notes detail the key findings of the conducted research. EHR, electronic health records; CVD, cardiovascular disease; BEHRT, Bidirectional EHR Transformer; SBP, systolic blood pressure; CI, confidence intervals.

**See the editorial comment for this article ‘Deep learning approach to unmask hidden salt effects in the era of artificial intelligence’, by A.J. Manolis and M.S. Kallistratos, https://doi.org/10.1093/eurheartj/ehad673.**

## Introduction

Paracetamol is the most commonly used analgesic worldwide and is recommended as a first-line treatment of pain for many acute and chronic conditions. To address issues of elevated risk of hepatotoxicity and low systemic bioavailability in oral administration, an effervescent formulation of the drug with sodium bicarbonate was launched into the market.^1,2^

However, since effervescent formulations include sodium, concerns have been raised that they may increase blood pressure (BP) and subsequently the risk of cardiovascular diseases (CVD) and mortality. While consequential if well-founded, there is little aetiological support for sodium bicarbonate–driven risk as opposed to the well-established sodium chloride and BP pathway.^3,4^ Nevertheless, observational studies using UK electronic health records (EHR) data have claimed that initiating sodium-containing paracetamol is associated with an elevated risk of high BP, incident CVD, and all-cause mortality.^5,6^ One study even showed that exposure to sodium-based paracetamol has a stronger association with all-cause mortality [hazard ratio (HR) ∼2.0] than vascular outcomes (HR ∼1.5).^6^ However, the tenuous biological support underpinning these observations raises concerns of the possibility of uncontrolled confounding. Indeed, conventional expert-guided confounder selection is prone to omitting important confounding variables.^7^ Given these concerns and the major clinical implications of the findings, we found it prudent to independently assess the associations.

We sought to employ a validated deep learning (DL) approach for hypothesis testing, which has recently shown promise for more comprehensive capturing of known and latent variables confounding association in the context of rich EHR data.^8–10^ Specifically, we used the Targeted Bidirectional EHR Transformer (T-BEHRT) model to investigate the association of sodium-based paracetamol vs. non–sodium-based formulations with systolic blood pressure (SBP), incident CVD, and all-cause mortality in UK EHR data.^8^ To enable direct comparison of the results with previous work, we replicated the design and modelling approaches of previous studies as closely as possible.

## Methods

### Study setting and population

We used UK EHR from Clinical Practice Research Datalink (CPRD), validated for population-based epidemiological research (protocol number: 16_049R).^11,12^ Previous research that we wished to independently replicate was conducted on The Healthcare Improvement Network (THIN) data set.^6^ Both CPRD and THIN utilize the Vision software system for EHR recording and provide access to various health data such as diagnoses, prescriptions, measurements, and demographic variables.^13,14^ However, given that THIN does not offer linkage to hospital and complete mortality records (i.e. with cause of death information), we note that we did not use these data sources in our main analyses using CPRD data in order to enable more direct comparison with previous works.^6^

Using a primary care EHR, linked with mortality data from the Office of National Statistics (ONS), we identified a cohort of 475 442 individuals. We included people between 60 and 90 years of age with at least one prescription of paracetamol between 1 January 2000 and 1 January 2014, aiming to replicate the approach in previous works.^6^ The index date (i.e. baseline) was defined as the date of the first paracetamol prescription. Replicating previous studies, patients with a recorded diagnosis of any type of cancer, previous CVD [i.e. heart failure, stroke, and myocardial infarction (MI)], and prior use of compound paracetamol (e.g. paracetamol with codeine) were similarly excluded.^6^ Cancer and CVD were identified using previously validated disease phenotyping methods, while compound paracetamol was identified by the CPRD ‘product code’ (i.e. native coding system for medications designed by the CPRD organization).^12^

### Exposures and outcomes

Identified with CPRD ‘product codes’, we compared paracetamol formulations containing sodium (i.e. formulations of ‘soluble’ and ‘effervescent’) as the exposure group with non–sodium-based formulations (i.e. formulations of ‘capsule’, ‘tablet’, and ‘oral suspension’) as the comparison group.

As over 96% of patients had paracetamol prescription for <1 year, similar to past work, the follow-up duration was set for 1 year.^6^ We investigated the association with three outcomes: (i) SBP as a continuous outcome for patients with SBP measurements (as recorded in CPRD), (ii) incident major CVD defined as a composite of MI, heart failure, and stroke, and (iii) all-cause mortality, identified by death in the ONS registry.^6^ Cardiovascular disease outcomes were identified using validated phenotyping methods for CPRD.^12^ To mitigate measurement error, SBP was calculated as an average value of the measurements taken in a 6-month window around the 1-year mark (i.e. between 9 and 15 months following the index date).^15,16^

### Statistical methods

We used T-BEHRT, a DL model shown to conduct less biased association estimation in the observational EHR setting.^8,9^ The T-BEHRT model is a neural network–based approach for causal inference that utilizes three components for robust association estimation: (i) a modified BEHRT encoder for modelling of both static and temporal EHR variables (Supplementary data online, *Figure S1*), (ii) a DL-driven tandem propensity score and outcome prediction framework, and (iii) a semi-parametric, doubly robust (DR) estimator for more accurate association estimation.

First, more generally, the encoder model is employed to condense high-dimensional minimally processed EHR data into a rich compact vector with a limited number of continuous values that represents patient health at baseline.^11,17,18^ While the original BEHRT model solely handles longitudinal data, the modified BEHRT encoder architecture enables handling of both: (i) longitudinal clinical encounters (i.e. diagnoses or medications) with annotations of age and calendar year of recording and (ii) static variables (e.g. sex and ethnicity), and represents them as embeddings (Supplementary data online, *Figure S1B*)—high-dimensional trainable vectors that allow representation of concepts in numerical form, for which similar concepts have similar numerical representations (e.g. embeddings for ‘stroke’ and ‘transient ischaemic attack’ are more similar than ‘stroke’ and ‘malaria’).^8^ Following embedding of input data, through ‘deep’ non-linear neural network modelling, the BEHRT encoder extracts latent features between input data embeddings beyond temporal proximity and outputs a single distilled vector for each patient representing baseline health (i.e. the ‘pool’ element in Supplementary data online, *Figure S1A*).^11,17^

Second, the patient representation vector is used as input to jointly learn the propensity score, the probability of being assigned the exposure of interest, and the outcome of interest.^19^ More specifically, the model learns to predict the assigned exposure status for each patient during propensity score learning. Simultaneously, the model is trained to predict the outcome given patient exposure status on the same patient representation vector (i.e. ‘Conditional Outcome 0’ and ‘Conditional Outcome 1’ in Supplementary data online, *Figure S1A*). Following training, the T-BEHRT model is applied to conduct propensity score and counterfactual prediction (i.e. prediction of an unknown potential outcome); for each patient, the (i) propensity score, (ii) the outcome as if the patient was given the non-exposure, and (iii) the outcome as if the patient was given the exposure are simultaneously predicted.^8,19^

Third, given recent advancements in semi-parametric DR estimators for reducing selection bias, these three predictions for every patient are inputted into one such DR estimator for more accurate association estimation.^20,21^ Specifically, cross-validated targeted maximum likelihood estimation (CV-TMLE) is utilized to ‘correct’ or update the risk prediction estimates for each exposure group by utilizing propensity score prediction to mitigate selection biases distorting estimation.^21^ With corrected risk estimates for each exposure group, risk ratio (RR) or other estimands of interest can be produced with 95% confidence intervals (CI).

In this study, the T-BEHRT model incorporated all primary care diagnosis and medication records prior to baseline for each patient, with attributions of age and calendar year of recording, as well as static attributes of sex and smoking status at baseline. The CV-TMLE estimation of mean difference (MD) in mmHg for SBP as a continuous outcome and RR for binary outcomes was conducted.^8,21^ Analysis was additionally conducted on patients with and without hypertension (identified by validated phenotyping methods) at index date.^12^ Further details concerning DL modelling are reported in Supplementary data online, *Supplementary Methods*.

To directly compare the findings from T-BEHRT with conventional modelling of previous reports, we also implemented a two-stage propensity-based statistical regression modelling for incident CVD and all-cause mortality as outcomes.^6^ The propensity score was assessed with logistic regression, and inverse probability treatment weight was derived and utilized for log-binomial modelling for binary outcomes, incident CVD and all-cause mortality.^19^ Similar to past research, a total of 52 variables were adjusted, and appropriate imputation was conducted on missing continuous and categorical variables.^6^ Estimations of RR for incident CVD and all-cause mortality investigations in addition to associated 95% CI were derived from model coefficients. Lastly, the crude (unadjusted) association estimate was calculated for all three outcomes as a naive approach. Analyses of those with and without hypertension at the index date were additionally conducted.^12^ Details concerning conventional modelling are provided in Supplementary data online, *Supplementary Methods*.

We conducted three complementary and sensitivity analyses to investigate bias in our main analyses using T-BEHRT and to check the robustness of the findings. First, for shorter follow-up periods, one major source of bias is confounding due to incipient illness—the potential for the association between exposure and outcome to be driven by the causal pathway between subclinical illness and outcome as opposed to the exposure itself.^22^ Hence, in order to assess if incipient illness has a material impact on the associations, we re-analysed the data, excluding those who died in the first month.^22^ Specifically, the baseline remained unchanged, but only those who were living at the end of the first month were included in the study. This analysis was repeated for up to 6 months. Second, we conducted an analysis to explore the presence of any uncontrolled confounding due to the delayed recording of conditions that are known to be associated with mortality. To do this, we identified variables reported in the time interval between exposure and outcome that were most strongly associated with the exposure through unadjusted prevalence ratio (PR) modelling. Then, we conducted log-binomial adjusted modelling of the association between exposure and these individual variables, adjusted for sex, age, index of multiple deprivation, body mass index (BMI), region, ethnicity, alcohol status, and smoking status. Variables that demonstrated a non-null association with the exposure in the analyses were then additionally incorporated into the T-BEHRT modelling to assess any change in associations. Third, we pursued both of the aforementioned sensitivity analyses simultaneously to comprehensively mitigate the potential biases in the estimation of all-cause mortality risk. For these three sensitivity analyses, diagnosis records from secondary care were additionally included in T-BEHRT modelling.

Inspired by the design of similar studies and in order to (i) capture an enriched cohort of initiators of either paracetamol formulation and (ii) mitigate uncontrolled confounding due to short follow-up, we further replicated all aforementioned analyses of the association of paracetamol and the three outcomes in patients with at least two prescriptions of the sodium or non–sodium-based formulation for a follow-up of 5 years (i.e. a subgroup of our main cohort).^5^ A detailed explanation is provided in Supplementary data online, *Supplementary Methods*.

The REporting of studies Conducted using Observational Routinely-collected Data reporting guidelines were followed for this cohort study. The T-BEHRT source code is presented on the Deep Medicine research group codebase (https://github.com/deepmedicine).

## Results

A total of 475 442 eligible individuals were included in this study (Supplementary data online, *Figure S2*). A total of 460 980 and 14 462 patients were selected for the non–sodium-based and sodium-based paracetamol exposure groups, respectively. The median duration of exposure from index date for sodium-based paracetamol was 12 days (interquartile range [IQR]: [5, 16]) and for non–sodium-based, 14 days (IQR: [8, 17]). The mean follow-up time was 11 months for CVD and mortality outcomes. Patients’ characteristics by exposure categories are shown in *Table 1* with extended data presented in Supplementary data online, *Table S1*. The mean age at baseline was 74 (standard deviation: 8.6) years, and 64% were women. While many characteristics at index date including BMI, year of birth, and many disease and prescription patterns were balanced between both exposure groups, several (including smoking status, diabetes, hypertension, gout, dementia, and BP-lowering medications) were not, generally consistent with previous work.^6^ For the investigation of SBP as an outcome, 235 699 patients were included; baseline characteristics for this subset of patients were similar to the cohort for analyses of binary outcomes and comprehensively described in supplementary materials (Supplementary data online, *Table S2*).

**Table 1:** Baseline characteristics among patients initiating non–sodium-based or sodium-based paracetamol

Exposure	Non–sodium-based paracetamol	Sodium-based paracetamol
No. (%)	460 980 (97.0)	14 462 (3.0)
Age, yrs (SD)	73.7 (8.6)	76.1 (9.1)
Women (%)	296 190 (64.3)	10 342 (71.5)
Ethnicity (White) (%)	126 248 (27.4)	4060 (28.1)
IMD (SD)^a^	1.9 (1.4)	1.9 (1.3)
SBP (SD)^a^	141.2 (13.7)	139.4 (14.5)
BMI (SD)^a^	27.7 (4.3)	26.0 (4.0)
Smoking status^a^		
Current or former smoker (%)	252 522 (54)	5431 (37)
Never smoker (%)	208 458 (45)	9031 (62)
Alcohol status^a^		
Current or former drinker (%)	343 602 (74)	9221 (63)
Never drinker (%)	117 356 (25)	5241 (36)
Comorbidity at baseline		
CKD (%)	3757 (0.8)	86 (0.6)
Diabetes (%)	41 894 (9.1)	988 (6.8)
Hypertension (%)	118 022 (25.6)	2640 (18.3)
Arthritis (%)	140 161 (30.4)	2960 (20.5)
Gout (%)	16 239 (3.5)	297 (2.1)
Rheumatoid arthritis (%)	7477 (1.6)	238 (1.6)
Hyperlipidaemia (%)	35 861 (7.8)	737 (5.1)
Atrial fibrillation (%)	17 563 (3.8)	443 (3.1)
Fracture (%)	50 478 (11.0)	1596 (11.0)
Gastrointestinal bleeding (%)	5457 (1.2)	226 (1.6)
Reflux disease (%)	24 349 (5.3)	663 (4.6)
Gastritis (%)	15 600 (3.4)	474 (3.3)
Dementia (%)	9973 (2.2)	985 (6.8)
Medications used at baseline		
Statins (%)	112 124 (24.3)	2304 (15.9)
Blood pressure lowering (%)	216 574 (47.0)	5465 (37.8)
Anticoagulants (%)	21 102 (4.6)	570 (3.9)
Antiplatelet (%)	120 975 (26.2)	3612 (25.0)
Opioids (%)	139 717 (30.3)	3031 (21.0)
Glucose lowering (%)	6212 (1.3)	122 (0.8)
Insulin (%)	8357 (1.8)	209 (1.4)

SD, standard deviation; No., number; yrs, years; BMI, body mass index; SBP, systolic blood pressure; CKD, chronic kidney disease; IMD, index of multiple deprivation.

Imputed variables; the percentage of missing variables—alcohol status (54.6%), smoking status (36.9%), IMD (38.7), SBP (26.4%), and BMI (40.9%).

**Figure 1:**
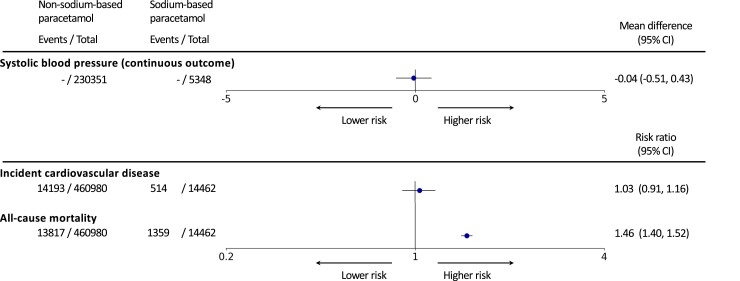
Association of sodium-based vs. non–sodium-based paracetamol and systolic blood pressure, incident cardiovascular disease, and all-cause mortality. From the left, Targeted-BEHRT estimates for each outcome are shown. Number of events and total number of patients in each exposure group are shown in the second and third columns. The forest plot and corresponding mean difference/risk ratio estimates are shown in the right-most column relative to the reference exposure, non–sodium-based paracetamol. The association estimate is plotted on linear and logarithmic scales for the mean difference in mmHg and risk ratio estimation, respectively. CI, confidence intervals.

**Figure 2:**
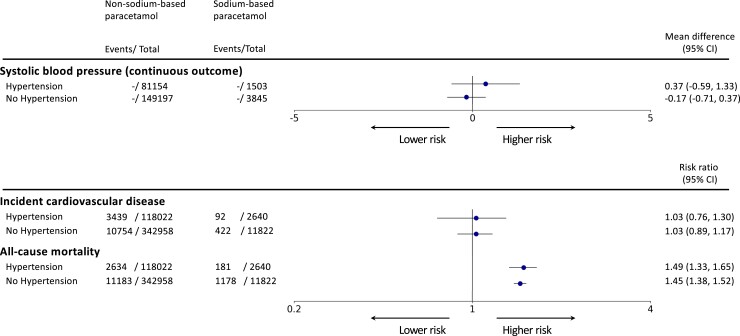
Association of sodium-based vs. non–sodium-based paracetamol and systolic blood pressure, incident cardiovascular disease, and all-cause mortality pressure for patients with/without hypertension at study entry. From the left, Targeted-BEHRT estimates for each outcome are shown by strata (hypertension, no hypertension). Number of events and total number of patients in each exposure group are shown in the second and third columns. The forest plot and corresponding mean difference/risk ratio estimates are shown in the right-most column relative to the reference exposure, non–sodium-based paracetamol. The association estimate is plotted on linear and logarithmic scales for the mean difference in mmHg and risk ratio estimation, respectively. CI, confidence intervals.

We observed no associations between formulations of paracetamol and SBP [MD: −0.04 (95% CI −0.51, 0.43)] or incident CVD [RR 1.03 (95% CI 0.91, 1.16)] (*Figure 1*). However, we found that sodium-based paracetamol is associated with an elevated risk of all-cause death [RR 1.46 (1.40, 1.52)] (*Figure 1*). Similar associations for three outcomes were found in a stratified analysis by baseline hypertension status (*Figure 2*).

In conventional modelling without adjustment, while there was no association in the overall analysis for SBP as the outcome, there was some heterogeneity in the subgroup analysis by hypertension status, with a rise in BP in those with a history of hypertension at baseline and a decrease in those without (Supplementary data online, *Figure S3*). Furthermore, a positive association with the risk of incident CVD events was observed in the analysis using conventional unadjusted and adjusted modelling (Supplementary data online, *Figure S3*). Also, for all-cause mortality as an outcome, results from unadjusted and two-stage adjusted modelling showed a greater magnitude of association compared with the results of the T-BEHRT model [RR 2.36 (2.29, 2.42)] (Supplementary data online, *Figure S3*).

### Sensitivity analyses

**Figure 3:**
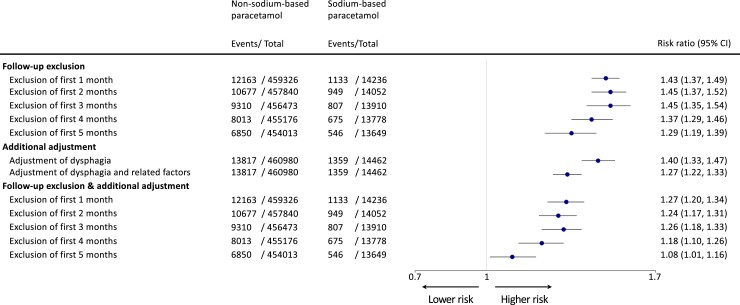
Forest plot of risk ratio estimates of T-BEHRT modelling with 95% confidence intervals for the association of paracetamol and all-cause mortality in sensitivity analyses. From the left, the type of sensitivity analyses is presented. Number of events and total number of patients in each exposure group are shown in the second and third columns. The forest plot and corresponding risk ratio estimates are shown in the right-most column relative to the reference exposure, non–sodium-based paracetamol. The association estimate is plotted on a logarithmic scale. CI, confidence intervals.

The absence of a material association with SBP and incident CVD raises the possibility of uncontrolled confounding as an explanation for the 46% increased risk of death in patients exposed to sodium-based paracetamol. To investigate this further, we first excluded those who had died in the first month of follow-up, repeated up to 6 months, and found the T-BEHRT association estimate attenuated by roughly 50% as compared to the association estimate for the entire cohort (*Figure 3*).

Then, we analysed factors associated with exposure and all-cause mortality recorded after the index date. This transgresses the usual principles of confounder selection, because post-baseline measures may be mediators or act as colliders, and adjustment for them may inappropriately attenuate or magnify associations.^23,24^ However, it is also likely that there will be delayed recording of relevant chronic conditions that could confound the association, meaning the conditions (or their prodrome) existed before recording. *Table 2* presents the first 10 variables ranked by the strength of the PR (exposure–variable association) with both unadjusted and adjusted modelling capturing similar directions of the association. Several neurocognitive and digestive tract disorders were identified as conditions associated with the exposure, and for these conditions, a long prodromal course indeed precedes diagnosis. Health service contact could be followed by both the initiation of sodium-based paracetamol and the eventual recording of the conditions in the patient’s EHR.

**Table 2:** Top 10 conditions identified after baseline with the largest difference in prevalence between the non–sodium-based and sodium-based groups

Disease	Prevalence in non–sodium-based paracetamol group (%)	Prevalence in sodium-based paracetamol group (%)	Unadjusted prevalence ratio	Adjusted prevalence ratio (95% CI)
Malignant neoplasm of oesophagus	0.15	0.85	5.83	5.34 (4.12, 6.93)
Pneumonitis due to solids and liquids	0.24	1.40	5.75	5.72 (5.00, 6.55)
Dementia in Alzheimer disease	0.18	0.75	4.27	3.78 (3.29, 4.33)
Dysphagia	0.4	1.70	4.23	3.39 (3.14, 3.67)
Alzheimer disease	0.75	3.08	4.11	2.76 (2.54, 3.01)
Multiple sclerosis	0.13	0.50	3.85	2.33 (1.90, 2.86)
Decubitus ulcer and pressure area	0.6	2.09	3.49	2.19 (1.87, 2.57)
Unspecified dementia	1.69	5.61	3.32	2.63 (2.46, 2.81)
Seizures	0.20	0.67	3.30	2.66 (2.35, 3.01)
Hemiplegia	0.24	0.69	2.92	2.67 (2.37, 3.02)

Unadjusted prevalence ratio: relative prevalence of a disease by ICD-10 code description in sodium-based paracetamol group divided by the same in the non–sodium-based exposure group (i.e. column 3 divided by column 2). Adjusted prevalence ratio: log-binomial modelling estimating association between exposure and variable (column 1) adjusting for baseline covariates.

95% CI, 95% confidence intervals.

Examination of these conditions revealed that they were all related to dysphagia, comorbid with dysphagia, or caused by dysphagia (*Table 2*).^25–30^ In light of this finding, additional adjustment of dysphagia with T-BEHRT modelling for all-cause mortality association estimation yielded a further modest risk reduction [RR 1.40 (1.33, 1.47)] (*Figure 3*) as compared to the main adjusted analysis [RR 1.46 (1.40, 1.52)]. Furthermore, when the same association was estimated with adjustment for all 10 variables (*Table 2*), the strength of the association was further attenuated [RR 1.27 (1.22, 1.33)] (*Figure 3*).

Next, we investigated a combination of the presented strategies: in addition to excluding patients who had died in the first month of follow-up, repeated up to 6 months, we estimated the association with additional adjustment of the 10 variables (*Table 2*). As the cut-off time was increased, the association estimate diluted commensurately. Investigating the association in patients alive 6 months into the follow-up period (‘Exclusion of first 5 months’ in *Figure 3*), T-BEHRT estimated RR: 1.08 (1.01, 1.16).

The final set of sensitivity analyses utilized T-BEHRT to investigate patients with at least two prescriptions of either exposure for a follow-up of 5 years. The findings were qualitatively similar to those of the main analysis (elaboration in *Supplementary Results*); no association was found with SBP and CVD outcomes, and for all-cause mortality as an outcome, T-BEHRT similarly captured an elevated risk of RR 1.18 (1.13, 1.23) (see Supplementary data online, *Table S3*). Accounting for factors recorded in the time between exposure and outcome (i.e. dysphagia and associated comorbidities shown in Supplementary data online, *Table S4* similar to those in the main analysis) mitigated excess risk towards unity [RR 1.07 (1.03, 1.12)] (see Supplementary data online, *Table S3*).

## Discussion

Utilizing a DL approach for analyses of rich EHR data, we found that sodium-based paracetamol had no association with SBP or incident CVD compared with non–sodium-based formulations (Structured Graphical Abstract). For all-cause mortality, our DL approach captured a biologically unexpected positive association in the main analysis; however, in sensitivity analyses, we found an attenuation of the association towards the null after considering factors distorting the association, but a weak association remained. By contrast, analyses based on conventional statistical models presented invariably positive associations of sodium-based paracetamol with all three outcomes.

Our analyses of the studied associations replicated previous research design but simultaneously addressed limitations of past works.^5,6^ Our study replicated past works as closely as possible in terms of patient selection, the exposure definition, the follow-up period definition, and adjusted modelling.^5,6^ As a result, both our selected cohort and that of previous research were similar in terms of exposure group imbalance (3.3% of patients initiating sodium-based paracetamol in research by Zeng *et al.* and 3.0% in our work) and while expansive variable selection was conducted in previous works (i.e. >50 variables), the fundamental issues of expert-guided confounder selection remained.^5,6^ These studies adjusted for baseline variables known to confound the association, but it seems that others unknown to experts have been omitted, thereby potentially biasing estimation. As an example, dementia was simply overlooked as a potential adjustment variable.^5,6^ We show that this variable, which is established to be independently associated with mortality, is clearly imbalanced between exposure groups^31^ (*Table 1*). This major omission might still be relatively easy to unveil, but it indicates that there may be several other unadjusted confounders distorting the paracetamol–mortality relationship. In such scenarios, the use of data-driven models such as T-BEHRT on large EHR with recorded information about known and latent confounders has an advantage. The T-BEHRT does not rely on conventional confounder selection and instead extracts confounders from minimally processed EHR more comprehensively than expert-guided approaches, as shown in previous simulation and observational studies.^8,9^ This could explain why a null association was found between exposure and cardiovascular outcomes.^8,9^

Although all methods used in this study demonstrated that sodium-based paracetamol was associated with a higher risk of all-cause mortality, sensitivity analyses using T-BEHRT illuminated that the conventional cohort study design might have limitations.^6^ Like conventional statistical models, T-BEHRT does not conventionally adjust for covariates measured after the index date. However, if important confounders that are clearly acting before the index date are not included because they have not been assessed—such as in the situation in which the prescription is precipitated by the medical encounter that eventually leads to the identification of what was a pre-existing condition—serious bias will be left unaccounted for. Specifically, apart from the confounders that typically precede the index date, our exploratory analyses additionally identified several conditions formally recorded after the index date that could reflect ongoing processes that confound the association. Given that issues of delayed diagnosis and, hence, recording of conditions in administrative EHR data are known, the PRs in *Table 2* are definitely not plausible estimates of an effect of using sodium-based paracetamol on these conditions (i.e. dysphagia and related conditions) but rather more plausibly relate to prodromal symptomatology influencing the prescription. Certainly, swallowing difficulty is one major indication for the selection of sodium-based formulations of paracetamol over its standard formulations.^28,30,32,33^ In a study with a relatively short follow-up duration, symptoms of dysphagia or its underlying causes (e.g. dementia or multiple sclerosis) might have a delayed capture in the EHR (Supplementary data online, *Figure S4*).^25–29,34,35^ Hence, adjustment of confounding by indication by dysphagia and related conditions (allowing for delayed recordings after the index date) led to a substantial weakening of the association between sodium-based paracetamol and all-cause mortality [RR 1.08 (1.01, 1.16)].

What is the explanation for the lack of an observed association between sodium-based paracetamol and BP or CVD? One potential reason is that the exposure duration was relatively short for the effect to manifest. Additionally, recent randomized evidence has demonstrated that regular daily use of non–sodium-based paracetamol increases SBP by roughly 5 mmHg compared with placebo.^36^ Hence, given the short duration of exposure in both groups, the iatrogenic risk caused by sodium in addition to paracetamol may be indifferentiable from the overall risk associated with paracetamol alone. Another possible explanation is that while sodium chloride is an established risk factor for elevated BP and CVD, sodium bicarbonate (i.e. the specific compound present in sodium-based paracetamol) does not cause a rise in BP. In an early investigation of pressor effects, chloride was found to be the primary source of SBP elevation while non-halide salts (e.g. sodium bicarbonate) demonstrated an alternate pressor effect.^4,37^ Subsequent randomized evidence confirmed this and curiously showed that sodium bicarbonate may potentially even reduce SBP in normotensive and hypertensive patients.^3,38^ Irrespective of the underlying cause, our study suggests that the current patterns of use of sodium-based paracetamol are not associated with excess risk of CVD.

Especially in multimorbid elderly patients, pain is a common symptom of many conditions. Inappropriate clinical recommendations derived from biased estimations of risk will limit access to one of the few pain management options available.^28,32^ The immaterial associations with CVD and SBP as outcomes should help mitigate concerns regarding the effects of sodium-based paracetamol. In the investigation of all-cause mortality, the strong positive association estimate was found to likely manifest from various biases as opposed to exposure. Nevertheless, an independent randomized investigation of the effects of sodium-based paracetamol on the studied outcomes compared to non–sodium-based formulations would be prudent to validate the findings.

### Strengths and limitations

In terms of strengths, first, we conducted the study under two different study designs to ensure the robustness of the findings. The extended analyses of patients with at least two prescriptions of a formulation and the longer follow-up period allowed for more nuanced analyses of combinations of cardiovascular endpoints in addition to further analyses of all-cause mortality and SBP as outcomes. Second, the T-BEHRT has been shown in both simulation and routine EHR data investigations to more accurately estimate association direction and strength by capturing known and latent confounders in comprehensive EHR, an asset in our approach not available in more conventional modelling approaches.^8–10^ In cohorts with heightened risk or for which little about confounding is understood, the DL approach provides better adjustment as confounder selection is model-driven. Third, multiple sensitivity analyses of all-cause mortality outcome enabled a more comprehensive understanding of the biases prevalent in this observational study. Specifically, follow-up exclusion analyses demonstrated the issues with a short follow-up period; alternatively, analysing all-cause mortality in a 5-year follow-up independently addressed these issues. Additionally, an inspection of variables recorded after the time of exposure illuminated how unadjusted confounding was present and was distorting estimates for the association with all-cause mortality. Fourth and lastly, we implemented conventional models to appropriately compare directly against our DL approach in order to replicate prior work.^5,6^

Our study also has some limitations. First, as over-the-counter prescriptions of paracetamol are not recorded in the CPRD, our study cannot be generalized to casual users of paracetamol. However, it is clinically unlikely that those prescribed with one formulation of paracetamol would procure over-the-counter medication of the other. Additionally, we mitigated cost-related opportunity biases by only including those at least 60 years of age at the index date as National Health Services in the UK offers many healthcare services at zero cost for these patients. As a result, any cost-related barriers to accessing formulations of paracetamol would be minimal, ultimately disincentivizing over-the-counter purchase for both exposure groups and further ensuring a less biased assessment of the studied associations. Second, further to the point of prescription usage, medication dispensation or retrieval information is not offered in CPRD; hence, prescription data may not be reflective of the actual usage of relevant medications. Third, CPRD, as an administrative data set, has known issues of recording biases and measurement errors. Incipient diseases at baseline due to the latent nature of conditions hampered the estimation of treatment effect in our study. A known issue in administrative data sets like CPRD is that often ‘milder’ or sub-clinical stages of conditions (e.g. incipient cancer) are difficult to precisely track in clinical records.^39^ However, appropriately accounting for these limitations in sensitivity studies can offer a more complete image of the various assumptions and biases at play. Fourth, SBP recordings are often known to be susceptible to measurement error; however, as suggested by previous research, summary measures using averaging are a validated method of alleviating distorted conclusions due to measurement error issues.^15,16^ Fifth, more precise time-to-event modelling of the outcome and patient censoring are necessary for association analyses. However, because survival DL modelling has not been appropriately developed for association estimation, nuanced evaluation of risk with time-to-event DL modelling should be explored in future studies. Sixth, over-adjustment (collider adjustment and M-structure biases) is a notable issue; however, empirical research has demonstrated that conditioning on all pre-treatment variables is still the optimal course of adjustment.^40,41^ Furthermore, clear index date definition and strict inclusion of pre-treatment EHR only up to index date further alleviates such issues. Seventh, while T-BEHRT has some advantages in how known and latent confounding are captured, we cannot rule out bias from unmeasured confounding. Specifically, in the study of all-cause mortality as an outcome, it is well established that bias due to confounding by indication cannot be fully resolved by confounding adjustment methods.^42^ However, in this work, we have tried to demonstrate through numerous orthogonal analyses in a triangulation framework that excess death due to sodium-based paracetamol captured in our work is likely due to residual confounding.^43^

## Conclusions

Using a DL approach for causal inference in the observational setting, we found that sodium-based paracetamol has no material association with the outcomes of SBP and incident CVD compared with non–sodium-based alternatives. In the case of all-cause mortality as the outcome, a weak association remained that could not be fully explained. In general, our findings do not substantiate the recommendation of issuing a warning for the prescription of sodium-based paracetamol, in particular, given the lack of an alternative for patients who suffer from dysphagia or related conditions.

## Supplementary Material

ehad535_Supplementary_Data

## Data Availability

Regarding accessibility of Clinical Practice Research Datalink (CPRD) data, https://www.cprd.com/primary-care explains: ‘Access to data from CPRD is subject to a full licence agreement containing detailed terms and conditions of use. Patient level datasets can be extracted for researchers against specific study specifications, following protocol approval from the Independent Scientific Advisory Committee (ISAC)’. Thus, restrictions apply to the availability of these data, which were used under licence for the current study, and so are not publicly available. More details of the data and data sharing are found on the CPRD website (https://www.cprd.com). The Targeted-BEHRT source code can be found on the Deep Medicine research GitHub site (https://github.com/deepmedicine).

## Pre-registered Clinical Trial Number

None supplied.
