# Supplementary material for: Sodium-based paracetamol: impact on blood pressure, cardiovascular events, and all-cause mortality
Source: Eur Heart J. 2023 Aug 23;44(42):4448–57. doi: 10.1093/eurheartj/ehad535 (PMC10635668; doi:10.1093/eurheartj/ehad535)
Supplement: ehad535_Supplementary_Data [file ehad535_supplementary_data.zip › Supplementary_Revised.docx]

*Sodium-based paracetamol: impact on blood pressure, cardiovascular events, and all-cause mortality.*

*Corresponding author: Dr. Shishir Rao, MSc, DPhil*

Table of Contents

[Supplementary Methods 2](#_Toc129257708)

[Supplementary Results 4](#_Toc129257709)

[Supplementary Figures 5](#_Toc129257710)

[Supplementary Tables 9](#_Toc129257711)

[References 17](#_Toc129257712)

## Supplementary Methods

**Supplementary Methods: Deep learning modelling**

The deep learning model, Targeted Bidirectional Electronic Health Records Transformer (T-BEHRT) was applied for the estimation of risk ratio (RR) for the association between paracetamol and cardiovascular outcomes/all-cause mortality and mean difference (MD) for systolic blood pressure as a continuous outcome.

Minimally processed EHR was included for adjustment in the T-BEHRT model. In order for input variables to be machine readable, the diagnoses records from primary care in the Read code format were mapped to ICD-10 format for consistency with later, downstream sensitivity analyses, in which secondary care records in the native ICD-10 format was used. This mapping to the ICD-10 code format led to a total of 1,497 disease codes. Furthermore, the medication codes in CPRD “product code” format were mapped to 386 codes in the British National Formulary (BNF) coding format. Next, we extracted smoking status at baseline (current, former, never a smoker) as last known status in past 12 months prior to baseline in addition to patient sex for inclusion as static variables in the T-BEHRT framework.

The T-BEHRT model is a deep learning framework for causal inference that utilises four seminal advances in the fields of deep learning, causality analyses, and semi-parametric statistics. First, T-BEHRT makes use of a modified BEHRT feature extractor for modelling of both static and temporal variables ^1^ (**Supplementary Figure S1A**). BEHRT, is a notable model that has achieved superior predictive performance on a range of EHR related prediction tasks ^2^. Static variables can be included in T-BEHRT as categorical (including binary) and continuous values. If categorical, the values will be represented by a 2-dimensional embedding matrix, with each possible categorical value, as an individual vector in said matrix ^1^. Temporal clinical encounters – i.e., diagnoses or medications – are represented in a similar matrix. Age and calendar years are also included as categorical variables and each have their own embedding matrices. The temporal encounter combined with age and calendar year of recording gives a more holistic summary of the EHR data point. More importantly in the framework of causality, the T-BEHRT model can adjust for disease and medication in a more nuanced way adjusting for time-varying effects of confounding variables (**Supplementary Figure S1B**).

Second, the model utilises a novel unsupervised learning regime to better capture latent confounding in the input EHR. Masked EHR modelling (MEM) is implemented to extract rich latent representations from the static and temporal inputs in parallel with propensity/outcome risk prediction. MEM, in gist, is an unsupervised learning method that first involves randomly masking some input encounters (or static variables), and then forces the model to predict the identity of the masked data. For example, perhaps in the sequence of encounters presented in **Supplementary Figure S1B**, the encounter “D3” is masked. The model, will be forced to predict the masked token using contextualised data temporally arranged around the “D3” encounter. In this way, the model can better capture pre-treatment variables that are confounding variables ^1,3^. In simulation exercises, this unsupervised objective has been shown to consistently estimate causal effect more accurately than both statistical and deep learning benchmarks ^1^.

Third, the model predicts jointly, the propensity score and the conditional outcomes given exposure status. In cross-validation framework training, the propensity score is trained on factual data (i.e., known exposure status) and the conditional outcome prediction is trained on the factual outcomes (shown in **Supplementary Figure S1A** following “pool”state) ^3^. For those patients who are in exposure group, only the outcome given exposure is predicted and hence, learned. For those in the non-exposure group, only the outcome given the non-exposure is predicted and hence, learned. In this way, the model conducts multi-task learning to capture features that are important for prediction of exposure and outcome; these are patently, confounding latent features extracted from the raw EHR input variables^4^. In testing, the model is applied on the test fold and predicts (1) propensity score, (2) outcome given exposure, and (3) outcome given non-exposure for all patients. In this way, counterfactual prediction is conducted; all three predictions are collected for estimation of the estimand in question (e.g., risk ratio).

Lastly, semi-parametric, doubly-robust estimators have been recently finding success in mitigating selection and finite sample estimation biases and thus better modelling causality. T-BEHRT utilises this doubly-robust estimation to further reduce bias. Deep learning portion of T-BEHRT modelling predicts risk and propensity score, and downstream, second-stage cross validated targeted maximum likelihood estimation (CV-TMLE) updates the risk utilising propensity score prediction mitigating selection biases distorting risk estimates ^1^. Patients with propensity scores between 0.03 and 0.97 were kept in the study ^1^. This feature of the T-BEHRT modelling was developed in the original methods publication introducing the model. Since the model was found to conduct least biased risk ratio estimation in simulated data experiments as compared to benchmark statistical and deep learning models with these hyperparameters, the same trimming probability thresholds (i.e., filtering those with <0.03 and >0.97) were borrowed for use in this work^1^.

**Supplementary Methods: Risk ratio estimation for T-BEHRT model**

Risk ratio (RR) and mean difference (MD) was estimated with respect to the reference class, the non-sodium based paracetamol exposure. T-BEHRT was first trained to predict exposure and outcome risk with k-fold cross validation (k=5) implemented for training and testing ^1^. Risk and propensity score predictions were collected across the 5 test sets and pooled, and CV-TMLE was implemented on the pooled estimates to further reduce bias in estimation of RR and MD. Furthermore, 95% confidence intervals were derived for all estimates ^1^.

**Supplementary Methods: Conventional modelling**

In order to appropriately conduct conventional modelling similar to past works, two-stage modelling was implemented for the conventional approach. In the first stage of the approach, the propensity score was assessed with logistic regression; inverse probability treatment weights (IPTW) were derived from the predictions of propensity score for each patient. In the second stage, the IPTWs were utilised as patient-wise weights in log-binomial modelling for binary outcomes, incident CVD and all-cause mortality. For both outcomes, the log-binomial models were regressed on the exposure variable^3^. The RR and associated 95% confidence intervals were derived with appropriate transformation (i.e., exponentiation) of the coefficient for the exposure variable in the log-binomial model for each outcome.

In terms of adjustment, the same predictors from Zeng et al were incorporated in our first stage logistic regression modelling with the exception of the Townsend Deprivation Index, which was replaced with the Index of Multiple Deprivation (IMD)^5^. For deriving baseline body mass index (BMI) and SBP (solely for comprehensive characterisation of patient health at baseline), the average of the measurements recorded in the 36 months before baseline were used to mitigate measurement error^6^. Multiple Imputation by Chained Equations (MICE) was conducted on missing continuous and categorical variables. 15 iterations of MICE were conducted.

**Supplementary Methods: Sensitivity analyses of enriched cohort (at least two prescriptions/5-year follow-up)**

Furthermore, inspired by the design of similar studies and in order to (1) capture an enriched cohort of initiators of either paracetamol formulation and (2) mitigate confounding by reverse causation, we pursued analyses of the association of paracetamol and the three outcomes in patients between 60 and 90 years of age between January 1 2000 and January 1 2010 with at least two prescriptions of the sodium or non-sodium formulation for a follow-up of five years (i.e., a subset of our main cohort)^7^. SBP, cardiovascular outcomes, and all-cause mortality were studied outcomes. For investigation of SBP as outcome, similar to main analyses, patients with SBP were included for analyses; SBP was calculated as an average value of the measurements taken in a 6-months window around the 5-year mark (i.e., between 57 and 63 months following baseline)^6^. For the investigation of the cardiovascular endpoints as outcome, combinations of the individual cardiovascular endpoints, heart failure, MI, stroke, and cardiovascular death were investigated. For the investigation of all-cause mortality as outcome, the analysis into exposure and outcome associated factors recorded after index date as previously introduced in **Methods: Statistical methods** were pursued using T-BEHRT modelling. For all of these analyses, diagnoses records from both primary and secondary care, medication records, and mortality records were used for T-BEHRT modelling and outcome ascertainment.

## Supplementary Results

**Supplementary Results: Analyses of enriched cohort (at least two prescriptions/5-year follow-up)**

The cohort for analyses consisted of 226,674 (220,621 sodium paracetamol/4,053 non-sodium paracetamol) for analyses of binary outcomes and 89,816 (88,388/1,428) patients for analysis of the SBP outcome. T-BEHRT captured a null effect measure across the investigations of SBP as a continuous outcome and combinations of individual cardiovascular endpoints (**Supplementary Table 3**). Similar to the main analysis, the T-BEHRT captured an elevated risk of all-cause mortality (1.18 [1.13, 1.23]); analyses into factors recorded in the time between exposure and outcome (i.e., dysphagia and associated comorbidities) were similar to those found in the main analysis (**Supplementary Tables 4)**. Accounting for these factors mitigated excess risk towards the line of parity (1.07 [1.03, 1.12]) (**Supplementary Table 3**). The results were qualitatively similar to estimates in main analysis.

## Supplementary Figures


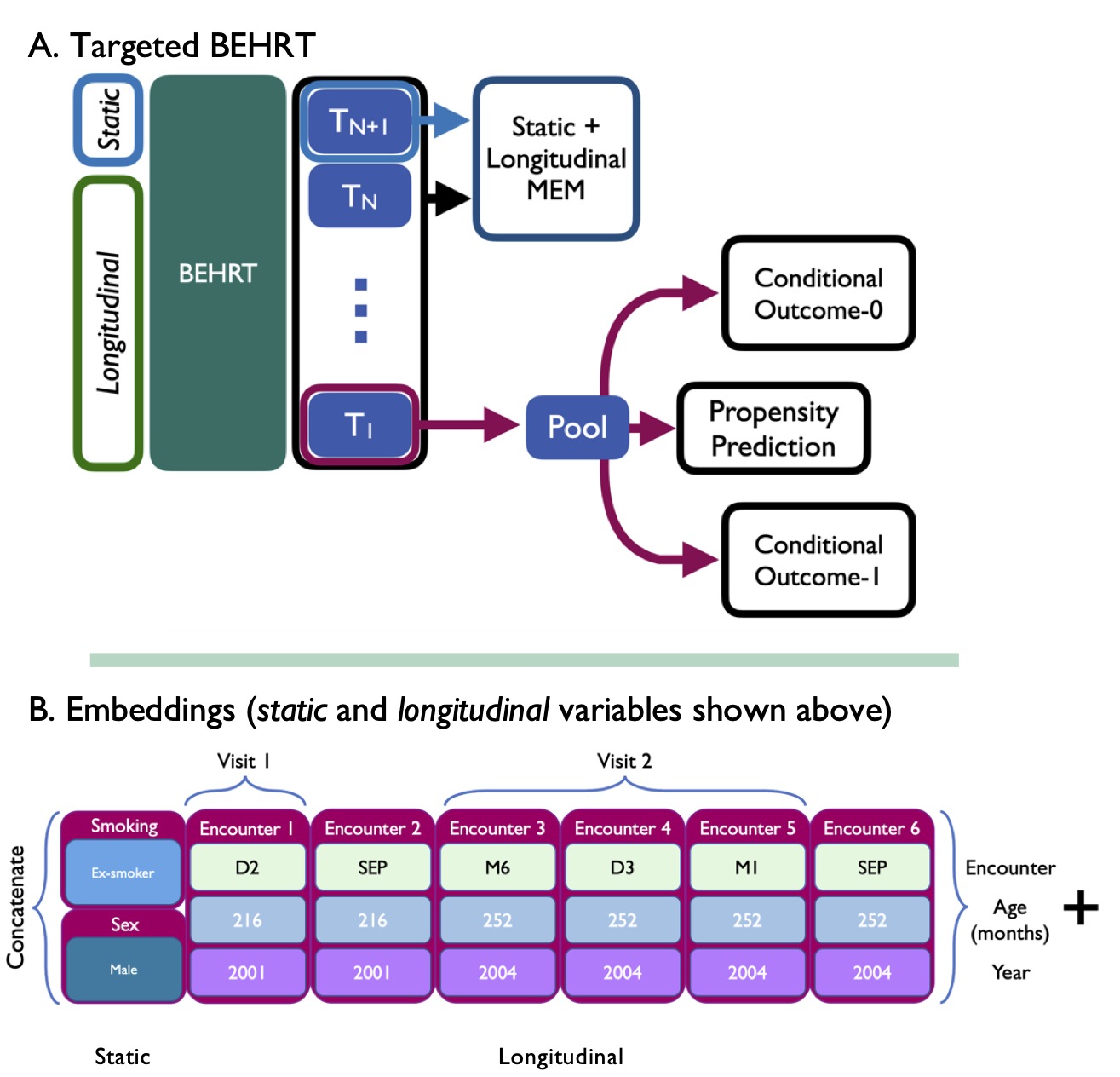


**Supplementary Figure S1**: T-BEHRT model architecture (A) and embedding design (B). (A) shows the static and longitudinal input, BEHRT feature extractor, the latent outputs for every clinical encounter (outputs T_1_ to T_N+1_) and the tasks for the models: (1) Masked EHR modelling (MEM), (2) propensity score prediction, (3) conditional outcome prediction (given exposure=0 or the reference group), (4) conditional outcome prediction (given exposure=1 or the intervention group). (B) shows the embedding structure. The embeddings include the static and longitudinal embedding structure. The diagnoses (e.g. D2, D8) and medications (e.g. M1) are fed into the model with the appropriate timestamp (age in months and calendar year) of recording. The embeddings for the encounter, age, and year are summed. The SEP element is a separator element used to inform the model that one visit has ended and another has started. The static attributes are similarly represented in high-dimensional embeddings and concatenated to the longitudinal data structure. In, sum the embedding structure incorporates static and longitudinal data inputs. EHR: electronic health records; SEP: Separator; T-BEHRT: Targeted BEHRT; MEM: Masked EHR Modelling

**
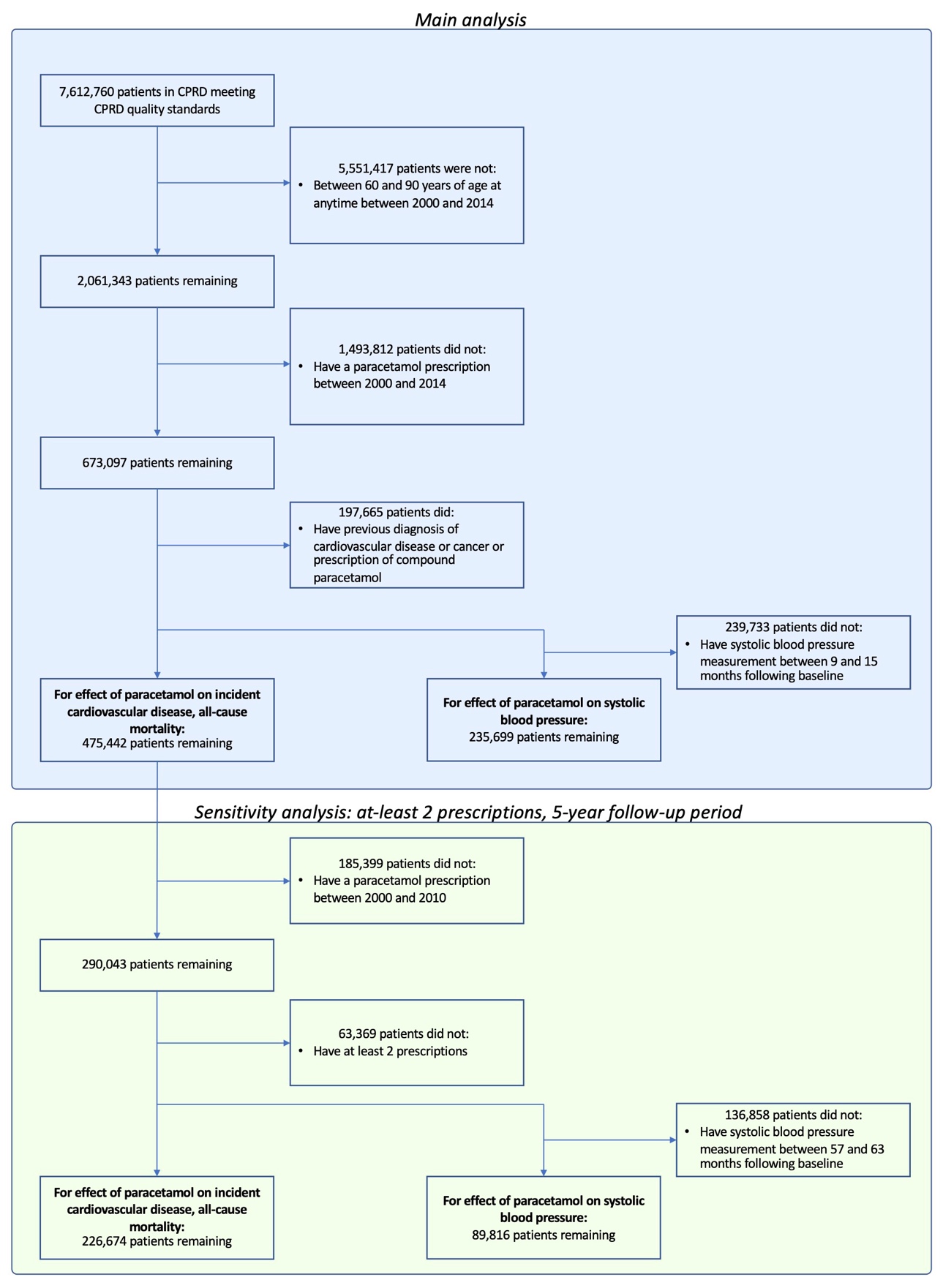
**

**Supplementary Figure S2 Flow chart of patient selection.**

*This is the flow chart to select eligible patients for this study. Main study is encapsulated in blue frame while the sensitivity analysis on an enriched subset of patients is encapsulated in green. CPRD: Clinical Practice Research Datalink.*


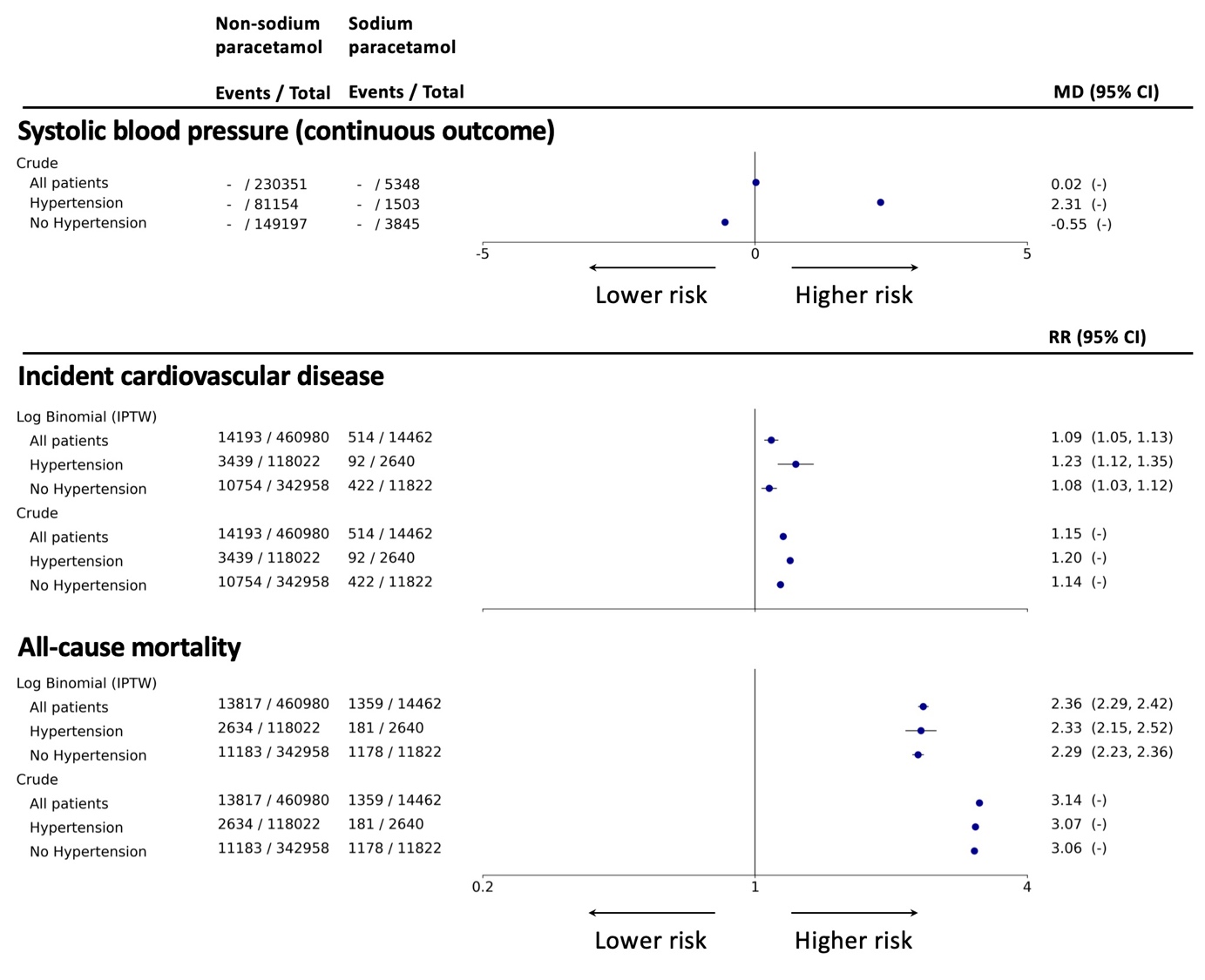


**Supplementary Figure S3 Association of sodium-based vs non-sodium-based paracetamol and systolic blood pressure, incident cardiovascular disease, and all-cause mortality with conventional modelling**

*From the left, the model and stratification status (all patients, stratified by hypertension status) is shown. Total number of patients in each exposure group is shown in second and third columns; Number of events is left blank. The forest plot and corresponding mean difference (MD)/risk ratio (RR) estimates are shown in the right-most column relative to the reference exposure, non-sodium paracetamol. The effect size is plotted on a linear and logarithmic scales for MD (mean difference in mm Hg) and RR estimation respectively.*


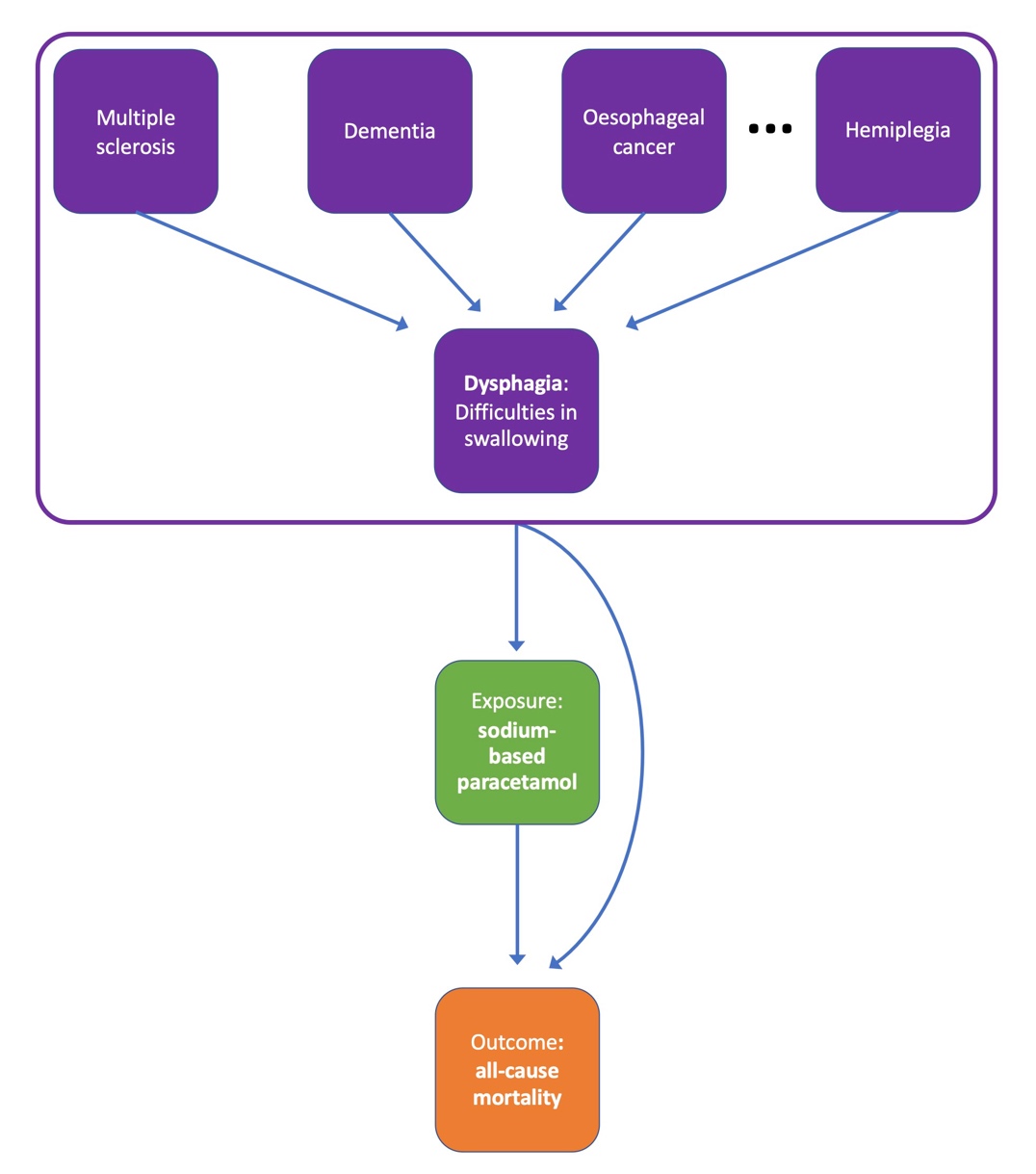


**Supplementary Figure S4 Diagram of confounding by dysphagia and related factors**

*The purple panel contains dysphagia and associated disorders connected to both exposure and outcome. The green box represents the exposure of sodium-based paracetamol, connected to outcome. The orange box is outcome of all-cause mortality.*

## Supplementary Tables

**Baseline characteristics for the investigation of risk of sodium-based paracetamol on all-cause mortality and incident cardiovascular disease as outcomes**

***Supplementary Table 1****: All baseline characteristics among patients initiating non-sodium based or sodium-based paracetamol*

| Exposure | Non-sodium | Sodium |
| --- | --- | --- |
| No. (%) | 460980 (97.0) | 14462 (3.0) |
| Age, yrs (STD) | 73.7 (8.6) | 76.1 (9.1) |
| Women (%) | 296190 (64.3) | 10342 (71.5) |
| Ethnicity (White) (%) | 126248 (27.4) | 4060 (28.1) |
| No. of GP visits (STD) | 3.3 (3.5) | 3.3 (3.7) |
| No. of secondary care visits (STD) | 1.3 (8.7) | 1.7 (6.7) |
| IMD (STD)* | 1.9 (1.4) | 1.9 (1.3) |
| YOB (STD) | 1933.4 (9.7) | 1930.2 (10.3) |
| SBP (STD)* | 141.2 (13.7) | 139.4 (14.5) |
| BMI (STD)* | 27.7 (4.3) | 26.0 (4.0) |
| Smoking status*: |  |  |
| Current or former smoker (%) | 252522 (54) | 5431 (37) |
| Never smoker (%) | 208458 (45) | 9031 (62) |
| Alcohol status*: |  |  |
| Current or former drinker (%) | 343602 (74) | 9221 (63) |
| Never drinker (%) | 117356 (25) | 5241 (36) |
| Disease at baseline: |  |  |
| CKD (%) | 3757 (0.8) | 86 (0.6) |
| Diabetes (%) | 41894 (9.1) | 988 (6.8) |
| Hypertension (%) | 118022 (25.6) | 2640 (18.3) |
| Arthritis (%) | 140161 (30.4) | 2960 (20.5) |
| Gout (%) | 16239 (3.5) | 297 (2.1) |
| Rheumatoid arthritis (%) | 7477 (1.6) | 238 (1.6) |
| Liver disease (%) | 1300 (0.3) | 41 (0.3) |
| PUD (%) | 6224 (1.4) | 176 (1.2) |
| Asthma (%) | 31659 (6.9) | 939 (6.5) |
| COPD (%) | 25775 (5.6) | 794 (5.5) |
| PAD (%) | 12949 (2.8) | 338 (2.3) |
| Epilepsy (%) | 3815 (0.8) | 213 (1.5) |
| Dementia (%) | 9973 (2.2) | 985 (6.8) |
| Depression (%) | 45231 (9.8) | 1324 (9.2) |
| Substance abuse (%) | 3260 (0.7) | 97 (0.7) |
| Hyperlipidaemia (%) | 35861 (7.8) | 737 (5.1) |
| Venous thromboembolism (%) | 18915 (4.1) | 471 (3.3) |
| Atrial fibrillation (%) | 17563 (3.8) | 443 (3.1) |
| Fracture (%) | 50478 (11.0) | 1596 (11.0) |
| Pneumonia (%) | 6471 (1.4) | 329 (2.3) |
| Fall (%) | 1227 (0.3) | 42 (0.3) |
| Gastrointestinal bleeding (%) | 5457 (1.2) | 226 (1.6) |
| Reflux disease (%) | 24349 (5.3) | 663 (4.6) |
| Gastritis (%) | 15600 (3.4) | 474 (3.3) |
| Medications at baseline: |  |  |
| Anticholinergics (%) | 243209 (52.8) | 7676 (53.1) |
| Statins (%) | 112124 (24.3) | 2304 (15.9) |
| Bisphosphonates (%) | 38965 (8.5) | 1194 (8.3) |
| Calcium (%) | 52365 (11.4) | 1839 (12.7) |
| Benzodiazepines (%) | 61521 (13.3) | 2254 (15.6) |
| Dementia (%) | 4241 (0.9) | 370 (2.6) |
| Antihypertensives (%) | 216574 (47.0) | 5465 (37.8) |
| Anticoagulants (%) | 21102 (4.6) | 570 (3.9) |
| Antiplatelet (%) | 120975 (26.2) | 3612 (25.0) |
| Anxiolytics and hypnotics (%) | 112258 (24.4) | 4033 (27.9) |
| Opioids (%) | 139717 (30.3) | 3031 (21.0) |
| Antipsychotic (%) | 85393 (18.5) | 3324 (23.0) |
| Steroids (%) | 83460 (18.1) | 2222 (15.4) |
| Nitrates (%) | 29985 (6.5) | 868 (6.0) |
| Loop diuretics (%) | 60549 (13.1) | 2094 (14.5) |
| Thiazide diuretics (%) | 129818 (28.2) | 3133 (21.7) |
| Potassium sparing diuretics (%) | 27226 (5.9) | 1013 (7.0) |
| Anti-diabetic (%) | 6212 (1.3) | 122 (0.8) |
| Calcium channel blockers (%) | 108754 (23.6) | 2528 (17.5) |
| ACE inhibitors (%) | 106362 (23.1) | 2361 (16.3) |
| Angiotensin receptor blockers (%) | 38717 (8.4) | 794 (5.5) |
| Beta blockers (%) | 103282 (22.4) | 2483 (17.2) |
| Oestrogen (%) | 65340 (14.2) | 1656 (11.5) |
| Insulin (%) | 8357 (1.8) | 209 (1.4) |
| H2 blockers (%) | 66324 (14.4) | 2018 (14.0) |
| Proton pump inhibitors (%) | 159288 (34.6) | 4379 (30.3) |
| DMARDs (%) | 7386 (1.6) | 198 (1.4) |
| Glucocorticoid (%) | 92014 (20.0) | 2391 (16.5) |

*%: percent; STD: standard deviation; No: number; Yrs: years; GP: general practice; YOB: year of birth; BMI: body mass index; SBP: systolic blood pressure; LDL: low-density lipoprotein; TC: total cholesterol; TG: triglycerides; CKD: chronic kidney disease; PUD: peptic ulcer disease; COPD: chronic obstructive pulmonary disease; PAD: peripheral artery disease; ACE: Angiotensin-converting enzyme; DMARDs: Disease-modifying antirheumatic drugs; *: imputed variables; the percentage of missing variables – alcohol status (54.6%), smoking status (36.9%), IMD (38.7), SBP (26.4%), BMI (40.9%).*

**Baseline characteristics for the investigation of risk of sodium-based paracetamol on systolic blood pressure as outcome**

***Supplementary Table 2****: All baseline characteristics among patients with systolic blood pressure measurements initiating non-sodium based or sodium-based paracetamol*

| Exposure | Non-sodium | Sodium |
| --- | --- | --- |
| No. (%) | 230351 (97.7) | 5348 (2.3) |
| Age, yrs (STD) | 73.7 (8.3) | 75.4 (8.9) |
| Women (%) | 149345 (64.8) | 3929 (73.5) |
| Ethnicity (White) (%) | 64540 (28.0) | 1618 (30.3) |
| No. of GP visits (STD) | 3.6 (3.6) | 3.6 (3.7) |
| No. of secondary care visits (STD) | 1.2 (8.3) | 1.5 (5.6) |
| IMD (STD*) | 1.9 (1.4) | 1.9 (1.4) |
| YOB (STD) | 1933.5 (9.3) | 1931.2 (10.1) |
| SBP (STD) | 142.4 (14.3) | 141.8 (15.6) |
| BMI (STD)* | 28.1 (4.6) | 26.6 (4.5) |
| LDL (STD) | 3.1 (1.4) | 3.1 (0.7) |
| TG (STD) | 1.6 (1.5) | 1.6 (0.7) |
| TC (STD) | 5.2 (1.7) | 5.2 (0.9) |
| Smoking status*: |  |  |
| Current or former smoker (%) | 122388 (53) | 2018 (37) |
| Never smoker (%) | 107963 (46) | 3330 (62) |
| Alcohol status*: |  |  |
| Current or former drinker (%) | 171599 (74) | 3496 (65) |
| Never drinker (%) | 58742 (25) | 1852 (34) |
| Disease at baseline: |  |  |
| CKD (%) | 2433 (1.1) | 41 (0.8) |
| Diabetes (%) | 29231 (12.7) | 555 (10.4) |
| Hypertension (%) | 81154 (35.2) | 1503 (28.1) |
| Arthritis (%) | 74113 (32.2) | 1301 (24.3) |
| Gout (%) | 9592 (4.2) | 133 (2.5) |
| Rheumatoid arthritis (%) | 3799 (1.6) | 99 (1.9) |
| Liver disease (%) | 605 (0.3) | 13 (0.2) |
| PUD (%) | 3041 (1.3) | 66 (1.2) |
| Asthma (%) | 16927 (7.3) | 439 (8.2) |
| COPD (%) | 12721 (5.5) | 304 (5.7) |
| PAD (%) | 7238 (3.1) | 149 (2.8) |
| Epilepsy (%) | 1689 (0.7) | 73 (1.4) |
| Dementia (%) | 3459 (1.5) | 207 (3.9) |
| Depression (%) | 22608 (9.8) | 518 (9.7) |
| Substance abuse (%) | 1360 (0.6) | 25 (0.5) |
| Hyperlipidaemia (%) | 22207 (9.6) | 411 (7.7) |
| Venous thromboembolism (%) | 9884 (4.3) | 182 (3.4) |
| Atrial fibrillation (%) | 10162 (4.4) | 198 (3.7) |
| Fracture (%) | 24039 (10.4) | 589 (11.0) |
| Pneumonia (%) | 3095 (1.3) | 107 (2.0) |
| Fall (%) | 611 (0.3) | 11 (0.2) |
| Gastrointestinal bleeding (%) | 2756 (1.2) | 81 (1.5) |
| Reflux disease (%) | 13139 (5.7) | 297 (5.6) |
| Gastritis (%) | 8211 (3.6) | 206 (3.9) |
| Medications at baseline: |  |  |
| Anticholinergics (%) | 123050 (53.4) | 2852 (53.3) |
| Statins (%) | 71388 (31.0) | 1220 (22.8) |
| Bisphosphonates (%) | 19423 (8.4) | 460 (8.6) |
| Calcium (%) | 25976 (11.3) | 706 (13.2) |
| Benzodiazepines (%) | 30821 (13.4) | 810 (15.1) |
| Dementia (%) | 1513 (0.7) | 81 (1.5) |
| Antihypertensives (%) | 139362 (60.5) | 2807 (52.5) |
| Anticoagulants (%) | 11607 (5.0) | 236 (4.4) |
| Antiplatelet (%) | 71042 (30.8) | 1476 (27.6) |
| Anxiolytics and hypnotics (%) | 55343 (24.0) | 1412 (26.4) |
| Opioids (%) | 70787 (30.7) | 1196 (22.4) |
| Antipsychotic (%) | 42913 (18.6) | 1142 (21.4) |
| Steroids (%) | 43083 (18.7) | 938 (17.5) |
| Nitrates (%) | 17495 (7.6) | 355 (6.6) |
| Loop diuretics (%) | 32194 (14.0) | 753 (14.1) |
| Thiazide diuretics (%) | 86469 (37.5) | 1749 (32.7) |
| Potassium sparing diuretics (%) | 14320 (6.2) | 366 (6.8) |
| Anti-diabetic (%) | 4436 (1.9) | 71 (1.3) |
| Calcium channel blockers (%) | 72588 (31.5) | 1369 (25.6) |
| ACE inhibitors (%) | 72549 (31.5) | 1301 (24.3) |
| Angiotensin receptor blockers (%) | 27605 (12.0) | 498 (9.3) |
| Beta blockers (%) | 65951 (28.6) | 1262 (23.6) |
| Oestrogen (%) | 34010 (14.8) | 731 (13.7) |
| Insulin (%) | 5742 (2.5) | 119 (2.2) |
| H2 blockers (%) | 34181 (14.8) | 842 (15.7) |
| Proton pump inhibitors (%) | 82365 (35.8) | 1763 (33.0) |
| DMARDs (%) | 3987 (1.7) | 93 (1.7) |
| Glucocorticoid (%) | 47660 (20.7) | 1015 (19.0) |

*%: percent; STD: standard deviation; No: number; Yrs: years; GP: general practice; YOB: year of birth; BMI: body mass index; SBP: systolic blood pressure; LDL: low-density lipoprotein; TC: total cholesterol; TG: triglycerides; CKD: chronic kidney disease; PUD: peptic ulcer disease; COPD: chronic obstructive pulmonary disease; PAD: peripheral artery disease; ACE: Angiotensin-converting enzyme; DMARDs: Disease-modifying antirheumatic drugs; *: imputed variables.*

***Supplementary Table 3:*** *Sensitivity analyses of patients with at least two prescriptions of either sodium/non-sodium paracetamol.*

| *Condition* | | | | | |  | *Events/Total* | |  | *Adjusted modelling* |
| --- | --- | --- | --- | --- | --- | --- | --- | --- | --- | --- |
| Stroke | HF | MI | CV death | ACM | SBP |  | Non-sodium paracetamol | Sodium paracetamol |  | T-BEHRT RR (95% CI) |
| • |  |  |  |  |  |  | 14709/220621 | 430/6053 |  | 0.93 (0.75 to 1.11) |
|  |  | • |  |  |  |  | 19030/220621 | 436/6053 |  | 0.92 (0.76 to 1.07) |
|  | • |  |  |  |  |  | 12218/220621 | 325/6053 |  | 0.86 (0.70 to 1.03) |
| • |  | • |  |  |  |  | 31464/220621 | 812/6053 |  | 0.91 (0.78 to 1.03) |
| • | • | • |  |  |  |  | 38241/220621 | 1004/6053 |  | 0.92 (0.84 to 1.00) |
| • |  | • | • |  |  |  | 34992/220621 | 1027/6053 |  | 0.96 (0.88 to 1.04) |
| • | • | • | • |  |  |  | 41649/220621 | 1225/6053 |  | 0.97 (0.89 to 1.06) |
|  |  |  |  | • |  |  | 24765/220621 | 1311/6053 |  | 1.18 (1.13 to 1.23) |
|  |  |  |  | • |  |  | 24765/220621 | 1311/6053 |  | Dysphagia adjustment:  1.15 (1.09 to 1.20) |
|  |  |  |  | • |  |  | 24765/220621 | 1311/6053 |  | Dysphagia + related comorbidities adjustment: 1.07 (1.03 to 1.12) |
|  |  |  |  |  |  |  |  |  |  |  |
| *Condition* | | | | | |  | *-/Total* | |  | *Adjusted modelling* |
| Stroke | HF | MI | CV death | ACM | SBP |  | Non-sodium paracetamol | Sodium paracetamol |  | T-BEHRT MD (95% CI) |
|  |  |  |  |  | • |  | -/88388 | -/1428 |  | -0.53 (-2.18 to 1.12) |

*RR: risk ratio; MD: mean difference; CV: cardiovascular; MI: myocardial infarction; HF: heart failure; ACM: all-cause mortality; SBP: systolic blood pressure; 95% CI: 95% confidence interval;* • *: analysed outcome.*

***Supplementary Table 4****: Top ten conditions identified after baseline with the largest difference in prevalence between the non-sodium based and sodium-based groups in analyses of patients with at least two prescriptions for 5-year follow-up.*

| Disease | Prevalence in non-sodium-based paracetamol group (%) | Prevalence in sodium based paracetamol group (%) | Unadjusted prevalence ratio | Adjusted prevalence ratio; (95% CI) |
| --- | --- | --- | --- | --- |
| Motor neurone disease | 0.20 | 0.97 | 4.92 | 4.58; (3.19, 6.56) |
| Alzheimer Disease (unspecified) | 1.95 | 5.72 | 2.93 | 1.99; (1.76, 2.24) |
| Pneumonitis due to inhalation of food and vomit | 0.89 | 2.54 | 2.87 | 2.84; (2.36, 3.41) |
| Multiple Sclerosis | 0.34 | 0.91 | 2.69 | 2.24; (1.64, 3.07) |
| Dementia in Alzheimer disease, unspecified | 0.43 | 1.06 | 2.48 | 2.32; (1.87, 2.87) |
| Malignant neoplasm of oesophagus | 0.51 | 1.17 | 2.32 | 2.45; (1.76, 3.41) |
| Decubitus ulcer and pressure area | 1.15 | 2.66 | 2.30 | 2.20; (1.91, 2.53) |
| Oesophageal obstruction | 0.43 | 0.99 | 2.29 | 2.06; (1.66, 2.56) |
| Other symptoms and signs concerning food and fluid intake | 0.11 | 0.25 | 2.28 | 1.84; (1.15, 2.95) |
| Dysphagia | 1.51 | 3.25 | 2.16 | 2.28; (2.02, 2.57) |

*Unadjusted prevalence ratio: relative prevalence of a disease by ICD-10 code description in sodium-based paracetamol group divided by the same in the non-sodium-based exposure group (i.e., column 3 divided by column 2); Adjusted prevalence ratio: log-binomial modelling estimating association between exposure and factor adjusting for baseline covariates; 95% CI: 95% confidence intervals.*

## References

1. Rao S, Mamouei M, Salimi-Khorshidi G, Li Y, Ramakrishnan R, Hassaine A, et al. Targeted-BEHRT: Deep Learning for Observational Causal Inference on Longitudinal Electronic Health Records. *IEEE Trans Neural Netw Learn Syst* 2022; 1–12.

2. Li Y, Rao S, Solares JRA, Hassaine A, Ramakrishnan R, Canoy D, et al. BEHRT: Transformer for Electronic Health Records. *Sci Rep* 2020; 10: 7155.

3. Rosenbaum PR, Rubin DB. The central role of the propensity score in observational studies for causal effects. *Biometrika*. Epub ahead of print 1983. DOI: 10.1093/biomet/70.1.41.

4. Zhang Y, Yang Q. An overview of multi-task learning. *Natl Sci Rev* 2018; 5: 30–43.

5. Zeng C, Rosenberg L, Li X, Djousse L, Wei J, Lei G, et al. Sodium-containing acetaminophen and cardiovascular outcomes in individuals with and without hypertension. *Eur Heart J*. Epub ahead of print 2022. DOI: 10.1093/eurheartj/ehac059.

6. Hutcheon JA, Chiolero A, Hanley JA. Random measurement error and regression dilution bias. *BMJ (Online)*; 340. Epub ahead of print 2010. DOI: 10.1136/bmj.c2289.

7. George J, Majeed W, Mackenzie IS, MacDonald TM, Wei L. Association between cardiovascular events and sodium-containing effervescent, dispersible, and soluble drugs: Nested case-control study. *BMJ (Online)*; 347. Epub ahead of print 2013. DOI: 10.1136/bmj.f6954.
